# Epistaxis kit ‒ a standardized approach to optimize emergency care

**DOI:** 10.1016/j.bjorl.2026.101782

**Published:** 2026-03-19

**Authors:** Luciano Lobato Gregorio, Eduardo Macoto Kosugi

**Affiliations:** Universidade Federal de São Paulo (UNIFESP), Escola Paulista de Medicina (EPM), Department of Otolaryngology and Head and Neck Surgery, São Paulo, SP, Brazil

Dear Editor,

We want to share a standardized approach implemented since 2013, designed to enhance the management of epistaxis within the Rhinology Division of the Universidade Federal de São Paulo – Escola Paulista de Medicina and the Emergency Department of Hospital São Paulo. This project, called the “Epistaxis Kit,” aims to improve the efficiency and effectiveness of care for patients presenting with nasal bleeding.

Even for experienced otolaryngologists, epistaxis continues to pose a clinical challenge. The stress and urgency of active bleeding, along with the anxiety of the patient and caregiver, can make clinical performance worse. Quick access to appropriate medications and instruments is essential to make doctors comfortable and patients safe. The Epistaxis Kit was made to facilitate quick, organized, and effective intervention, recognizing that delayed or poor management may result in possible complications.

Comparable to a “crash cart,” it puts together all necessary materials and medications into one easy-to-reach, readily accessible kit. Its contents include:

- Medications: Adrenaline, oxymetazoline, trichloroacetic acid (70–90%), lidocaine or neutotocaine (spray and gel), antibiotic/corticosteroid cream, distilled water, and saline solution.
- Nasal packing materials: Cotton, Pre-assembled glove finger gauze packs (various sizes), Foley catheters, gauze rolls, and commercial nasal packings (Merocel®, Rapid Rhino®).
- Lighting and instruments: Headlamps, nasal specula, bayonet forceps, nasal suction devices, bowls, domes, and scissors. Also, tapes, gauze, gloves, masks, caps, surgical gowns, and protective eyewear.
- may also include hemostatic agents: Gelfoam®, Surgicel®, Flo-seal®.

The kit has proven helpful not only in emergencies but also in inpatient and intensive care units, particularly for postoperative hemorrhage and spontaneous nosebleeds in hospitalized patients. Our initial experience showed a significant reduction in preparation time—from 10–15 minutes to under 2 minutes—and improved caregiver confidence during acute interventions.

We believe this innovative standardization can be readily adopted across both public and private healthcare institutions. Its implementation may reduce response times, improve clinical outcomes, and optimize resource utilization, contributing to costeffectiveness in hospital settings.

## ORCID ID

Eduardo Macoto Kosugi: 0000-0003-1318-0541

## Data availability statement

The authors declare that all data are available in repository.

## Declaration of competing interest

The authors declare no conflicts of interest.

